# The Identity of Organisms in Scientific Practice: Integrating Historical and Relational Conceptions

**DOI:** 10.3389/fphys.2020.00611

**Published:** 2020-06-17

**Authors:** Maël Montévil, Matteo Mossio

**Affiliations:** ^1^Institut d'Histoire et de Philosophie des Sciences et des Techniques (IHPST, UMR 8590), Université Paris 1 et CNRS, Paris, France; ^2^Centre Pompidou, Institut de Recherche et d'Innovation, Paris, France

**Keywords:** organization, genealogy, constraints, measurement, biological identity, variation, mathematical modeling

## Abstract

We address the identity of biological organisms at play in experimental and modeling practices. We first examine the central tenets of two general conceptions, and we assess their respective strengths and weaknesses. The historical conception, on the one hand, characterizes organisms' identity by looking at their past, and specifically at their genealogical connection with a common ancestor. The relational conception, on the other hand, interprets organisms' identity by referring to a set of distinctive relations between their parts, and between the organism and its environment. While the historical and relational conceptions are understood as opposed and conflicting, we submit that they are also fundamentally complementary. Accordingly, we put forward a hybrid conception, in which historical and relational (and more specifically, organizational) aspects of organisms' identity sustain and justify each other. Moreover, we argue that organisms' identity is not only hybrid but also bounded, insofar as the compliance with specific identity criteria tends to vanish as time passes, especially across generations. We spell out the core conceptual framework of this conception, and we outline an original formal representation. We contend that the hybrid and bounded conception of organisms' identity suits the epistemological needs of biological practices, particularly with regards to the generalization and reproducibility of experimental results, and the integration of mathematical models with experiments.

## 1. Introduction

Scientists often describe biological organisms as exquisitely complex objects. The adjective “complex” has various meanings, and one points to a difficulty in providing an adequate account of their identity, notably in modeling and experimental practices. What does organisms' identity refer to? As for any object, the identity of an organism designates what makes it what it is and, thereby, what makes it different from something else.

We can understand every conception of organisms' identity as spanning over a spectrum going from more stringent to more inclusive interpretations. At one end of the spectrum, the identity of an organism points to its *unicity*, i.e., the fact of possessing a unique set of properties, making it different from any other organism (and, a fortiori, from any other object). On the other end, the identity of an organism refers to its *individuation*, i.e., the fact of possessing those properties that allow drawing its boundaries and discriminating it from the surroundings. The reason why we take here individuation as the most inclusive interpretation of identity (among the many possible ones in the spectrum) is that even though organisms differ in many respects, we assume that they share a few (if not the very same) fundamental properties on the basis of which they can be isolated and recognized *as organisms*. Identity as unicity is often referred to as numerical or absolute, while identity as individuation—as well as for all possible intermediate interpretations—is relative, in the sense of only holding in relation to specific properties (Noonan and Curtis, 2018).

Each interpretation of identity in the spectrum provides criteria that generate a *reference class*. When understood as unicity, each identity class is supposed to contain only one organism; when understood as individuation, on the opposite side, a class should contain the largest number of (if not all) organisms. We understand more inclusive classes as being presupposed by more restrictive ones: in particular, the unicity of a given organism presupposes that it also meets the more general requirements for individuation. Furthermore, as philosophers commonly point out (see for instance Boniolo and Testa, 2012), the question of identity can be raised both at a given moment (“synchronic” identity or “who” question) and through time (“diachronic” identity or “persistence” question). Whatever interpretation of identity is adopted, one can investigate not only whether a given organism meets the criteria of membership to the reference class here and now, but also whether it keeps complying with them over time; the more the class is restrictive, the less it tolerates changes.

The choice of the interpretation of organisms' identity depends on the aim pursued. In science, moreover, interpretations and classes are not supposed to be merely arbitrary or practical groupings of objects: to be relevant, they should stem from theoretical conceptions and frameworks (Grimaldi and Engel, 2007). In evolutionary biology, notably, organisms are classified into several taxa, which in turn form a hierarchy of taxonomic ranks that includes the species, the genus, the family, up to life as a whole. These taxa are grounded in evolutionary theory (Lecointre and Le Guyader, 2006), and serve many purposes as eliciting further questions on evolutionary processes or providing tools for conservation biology (Godfray et al., 2004).

In this paper, we focus on the concept of organisms' identity that is relevant to experimental and modeling practices in Biology. Experimental practices require observing particular organisms. Yet, the knowledge that biologists usually try to obtain from their experiments is not supposed to be just about particular organisms but, instead, to hold for any other organism endowed with the *same* relevant properties. In particular, biologists need some theoretical justification for considering that several organisms are instances of the same experimental object, so as to distinguish the effects of experimental difference-makers from unrelated, spontaneous variations (Waters, 2007). In other words, experimental results obtained about a particular organism, or a few particulars, should apply to any other organism belonging to the same class. What is at stake is the generalizability of scientific knowledge and the related reproducibility of experimental results—the latter facing currently a major crisis, especially in biomedical research (Baker, 2016).

The complexity of biological organisms vis-à-vis identity is the acknowledged difficulty of treating particular organisms as instances of the same experimental object, and of subsuming them under the relevant classes (Agutter and Wheatley, 2004; Bookstein, 2009; Montévil, 2019a). Several reasons seem to play a role in explaining such difficulty.

The first reason is that, in both theoretical and empirical practices, scientists can only take into account a few aspects of biological organizations, understood here as the whole set of functions and processes constituting each organism. Typically, mathematical models only focus on some target features while neglecting many others, although such neglect does not rely on a clear theoretical justification and a systematic method. The same applies to experimental quantitative measurements, which are limited to only some aspects of the organisms under study.

A second reason, related to the previous one, is the strong coupling between biological organisms and their context. The context should be understood here in a comprehensive way, so as to include abiotic elements as well as other organisms, both participating in the determination of organisms' identity (Gilbert et al., 2012; Miquel and Hwang, 2016). Disentangling such a network of interactions requires understanding what matters and what does not when examining a specific phenomenon. For example, laboratory animals tend to have immunological properties that are different from those of wild animals because they usually experience a lower microorganisms biodiversity (Abolins et al., 2010).

A third reason is that contingent features that appeared throughout historical processes contribute to determining the properties of current organisms. In evolutionary theorizing, this idea corresponds to the “contingency thesis” (Beatty, 1995). Ontogenesis also conveys contingency, for example as a result of developmental plasticity (West-Eberhard, 2003). Organisms are contingent objects because they undergo continuous variations, and part of these variations last over time. Distinct individual organisms undergo different variations and generate new organisms that undergo further variations. Moreover, variations of organisms can also affect their context. Therefore, each organism results from such an intra- and cross-generation history of individual and contextual variations: in a word, organisms are historical objects (Montévil et al., 2016; Kauffman, 2019).

For all these reasons, an account of organisms' identity as experimental objects is a challenging task. Specifically, the challenge consists of adopting a conception of relative identity that generates one or several classes appropriate for the generalization and the reproducibility of experimental results. Such a conception would provide an operational tool for both empirical practices and mathematical modeling.

How is organisms' identity conceived in current biological practice? It seems to us that two broad theoretical conceptions can be distinguished. The first conception is *historical* or *genealogical*. Accordingly, a bat is a bat because all bats share a common ancestor, while other life forms do not (Lecointre and Le Guyader, 2006). Genealogy has here a twofold sense: a narrower one that maps onto reproductive relations; and a broader one that refers to the role of the past in determining the identity of a biological organism. In the latter sense, today Alice is Alice because she has been named so in the past, even though she has considerably changed over time. The second conception is *relational*. Biologists define organisms relative identity by referring to a set of relations between properties and traits that they possess. Following this strategy, a bat is a bat because it has the distinctive relations between properties and traits of bats.

As we will discuss, each conception is open to different interpretations of identity, going from more restrictive to more inclusive ones. For instance, evolutionary taxa also stem from a genealogical conception, but these classes are much more inclusive than the ones which are relevant for most experimental practices, where biologists deal with strains rather than species or higher ranks (see Montévil, 2019a, for a discussion and detailed examples). Importantly, the distinction between the genealogical and relational conceptions does not map onto the distinction between diachronic and synchronic identity, which means that each conception can be applied to characterize *both* the synchronic and diachronic identity of organisms.

Both conceptions are at work in experimental practices, and each of them has strengths and weaknesses. Genealogical strategies, we argue, enable scientists to consider *whole* organisms as identical without, however, making explicit the domain of validity of experimental results. In particular, it is unclear how much variation a set of genealogically connected organisms can undergo (during ontogenesis and across generations) while maintaining a relevant identity for a given experimental purpose. Relational strategies, in turn, make explicit their domain of experimental validity that, however, is restricted to the properties and relations explicitly taken into account. Organisms are relationally identical only insofar as it is possible to isolate such properties and to exclude any other aspects or changes that could (and actually do) make them different.

We can understand the relations between these two conceptions in different ways. One could favor the genealogical conception because it matches the historicity of biological organisms that emanates from the Darwinian theory of evolution. Alternatively, one could argue that the relational conception is the most fundamental one; its limited validity would be the mere effect of our (current) lack of theory and empirical knowledge. An example of the latter attitude (although not specifically addressing experimental practices) is Goodwin and Webster's relational theory of form changes that they take as a requirement to ground phylogenetic reasoning (Webster and Goodwin, 1996). As in physics' models of morphogenesis, the authors argue that genealogical categories (as homology) should stem from relational descriptions.

We advocate here a different view. We argue that biology requires combining genealogical and relational conceptions, with the support of an appropriate theoretical framework. The genealogical conception provides a procedure to select whole organisms as candidates to be subsumed into relevant identity classes. In turn, the relational conception – especially in an organizational version—provides explicit guidelines to understand the stability of biological organisms and, thereby, of the domain of validity of identity classes, notably in time. The main upshot of our analysis is a *hybrid* and *bounded* conception of organism identity. Organisms can be subsumed under hybrid identity classes that support the reproducibility and generalizability of experimental results. Nevertheless, the validity of identity classes for experimental practices is inevitably limited in time and space, which draws a fundamental difference between biology and other natural sciences, in particular physics and chemistry.

## 2. Contrasting Genealogical and Relational Conceptions of Identity

We describe in this section the two conceptions of organisms identity at work in experimental and modeling practices in biology, and we focus on their background epistemology. We aim at making explicit their respective strengths and weaknesses which, because of their complementarity, open the way to the elaboration of an integrated conception.

### 2.1. Genealogical Identity

A genealogical (or historical) conception of identity may take different forms. For instance, genealogical identity can be understood as the *preservation* of properties having occurred in the past. The version which is at work in biological disciplines conceives organisms' identity in terms of a more generic *connection* with the past. Several organisms are the same when they have a particular connection with the past in a historical process.

Historical identity is—unsurprisingly—at work in systematics, the discipline that elaborates the classification and taxonomy of biological organisms and whose results are used ubiquitously in biological practice. In systematics, particular organisms are considered as members of the same class if they belong to a monophyletic group, which includes only and all the descendants of a last common ancestor. How do systematics build classes? While the concept of genealogy comes from Darwin's theory of evolution, genealogies are usually not observable as such. For example, it is not possible to ascertain that a given fossil species is an ancestor of a current species. Instead, it is possible to show that a given specific fossil species is more closely related to a given current species than to another one. As a result, unlike the genealogy *stricto sensu*, phylogenetic groups are defined by their assessed genealogical proximity, and last common ancestors are theoretical specimens that biologists do not identify empirically (de Queiroz, 1992; Lecointre and Le Guyader, 2006; Lecointre, 2015).

The use of the genealogical conception of identity extends to day-to-day experimental practices across various biological disciplines. Biologists establish laboratory strains and usually run experiments on organisms coming from the same strain. By this practice, experimental biologists consider different individual organisms as hypothetically identical. For example, biologists assume that the properties of these organisms follow the same probabilistic distribution in statistical tests. When applying this conception, biologists do not exhibit a given set of observable properties that the organisms would share; instead, they build the identity class by referring to their shared recent origin. The “Methods” section of most experimental papers explicitly relies on this strategy.

Compared to the phylogenetic method of classification, the experimental practice is, at the same time, less conceptual and more operational. Experimental biologists do not estimate the genealogy by theoretical arguments based on similarities and hypotheses on evolutionary processes. Instead, they control genealogy empirically by letting the “ancestors” reproduce in laboratory conditions (Chia et al., 2005). Besides, the relevant identity classes at play in the experimental practice are often narrower than the taxonomic ranks. The latter often appear to be inadequate when trying to generalize experimental results. In the terms used above, we could say that experimental practices adopt a more restrictive interpretation of genealogical identity when compared to systematics.

Whatever interpretation is adopted, the genealogical strategy provides criteria that apply to both synchronic and diachronic identity of organisms. A group of organisms shares the same synchronic identity if they have a genealogical connection with a specific common ancestor. Likewise, each organism remains diachronically a member of the same class whatever difference (due to variation) appeared—or will appear—between it and the ancestor through time.

Identity classes built on genealogical conceptions (at least in the version discussed here) put no *principled* restrictions on the amount and nature of variations that each member of the class can undergo. The genealogical conception of identity can accommodate completely open futures, including the appearance of both structural and functional novelties, as well as radical changes of already existing structures and functions (Lecointre, 2015). Accommodating these novelties is a growing concern of theoretical biology (Montévil et al., 2016; Kauffman, 2019; Montévil, 2019b). Such inclusiveness is a strength of the genealogical conception of identity that enables biologists to accommodate the diversity of living organisms. For example, the “tetrapods” are organisms that have a common ancestor possessing four skeletal limbs. While most members of the class do share that trait, sub-classes such as snakes lost it. However, snakes remain part of the class since the definition refers to the common ancestor and not to the observable properties of the objects. This somehow paradoxical lesson can be generalized: no single observable trait or property has to be shared by a group of organisms being identical only by the reference to the past.

Let us mention one last aspect concerning genealogical strategies. In principle, ascribing a relative genealogical identity to a group of organisms requires estimating their genealogy and their connection to a common ancestor. However, in systematics, the common ancestor is not directly accessible and cannot be an empirical reference. Instead, biologists anchor a name to a specific individual organism called a “name-bearing type” that is the ultimate reference for this name (CZN International, 1999). Name-bearing types are not the common ancestor of a taxon but, instead, specimens that serve to define a name. The name is then extended to a group of organisms that includes the type and all the descent of a common ancestor, assessed by the methods of phylogeny (Lecointre and Le Guyader, 2006; Grandcolas, 2017). Experimental biologists can also obtain generations of organisms from an initial controlled group of organisms (although not necessarily from a specific individual common ancestor). Then the strain is defined by the reference to this group, often indirectly by the combination of the strain label and the name of the breeding institution. It is instructive to contrast these uses of particulars with the definitions in the International System of Units (Montévil, 2019a). These definitions rely on the physical theories that define reference units abstractly—they are invariants of the theory—and not on particular objects (such as the “prototype meter” that metrologists built afterward to instantiate these abstract definitions).

Although biologists do not use strains universally, organisms obtained in this way are widespread in experimental practices. Yet, what justifies the fact of subsuming them under taxonomic classes, and giving them names coming from systematics? The implicit hypothesis is that strains under control are subsets of taxonomic ranks: for instance, the strain “black 6” is supposed to belong to the systematic class of mice (*Mus musculus*). It also means that if we estimate the phylogeny of specimens of such a strain, including the initial group of organisms, they are more closely related to the member of the intended taxon, especially the name-bearing type, than to other taxons.

The genealogical conception of biological identity has several strengths. This conception allows ascribing an identity to organisms as *wholes* despite their relational complexity by building on the theoretical genealogies coming from the theory of evolution (even though it is not reducible to it, as just discussed). Furthermore, identity classes do not require conservation through time and leave the future open to indefinite variation. Historical identity is “invariant by reproduction”: if the parents are in a class, then the offspring will be in the same class because they share the same past, used as a reference.

In turn, genealogical identity suffers from significant weaknesses from the perspective of experimental practices, or applications such as medicine. While systematics aims at reconstructing the past and describing the present in light of the past, experimental practices investigate the relations between the parts of organisms, as well as between organisms and their surroundings. Because of these different goals, identity classes in systematics can include tetrapods that are such without having four limbs; in turn, empirical practices need classes that sustain reproducibility and generalizability of the results over a (hopefully large) group of organisms.

The source of the problem is the same that generates the strengths of historical definitions *per se*, i.e., the fact of being uniquely grounded in genealogical connections. Experimental biologists try to circumvent the problem by working mostly on groups of organisms having *close* ancestors, under the (implicit, but fundamental) hypothesis that genealogical proximity tends to go with organizational proximity: the closer individual organisms are in the genealogy, the less they tend to differ anatomically and functionally (Isaacs, 1986; Mogil et al., 1999; Montévil, 2019a). The main virtue of this precaution is that it does work to some extent in practice, which explains why it is widespread in empirical studies. Yet, no explicit justification of the underlying hypothesis is provided. As a result, the domain of validity for the experimental practice of genealogical identity classes is unknown, and there are no specifications about the rate and kind of variations (and, reciprocally, about the degree of similarity) that would threaten the membership to a given identity class.

### 2.2. Relational Identity

The relational conception of identity stems from a different epistemological stance. The description (and, in science, the theoretical determination) of an object mainly appeals to the *relationships* between its parts and constituents, as well as its relationships with other objects. Relations are understood as more fundamental and meaningful than non-relational aspects, notably because they have a stable form, amenable to mathematical descriptions such as equations. Moreover, the relational epistemology emphasizes that scientists ultimately observe objects via their relations with the measurement apparatus; therefore, relations can be seen as the starting point of experimental knowledge.

The relational epistemology pervades most natural sciences and especially physics. For example, although the electric charge seems to be an intrinsic property of objects, it is ultimately a quantity that describes how charged objects exert forces on each other: therefore it is grounded on relations^1^. According to the relational conception of identity, several objects are identical if they share the same relationships, and they are different if they do not. For example, all electrons are identical because they have the same relations with other objects (i.e., the same interactions), described by equations^2^. Similarly, a group of organisms belongs to the same identity class if they share a given set of relational properties.

What relations are relevant in the biological domain? After all, one may argue that genealogy is also a relation. In fact, what matters from a relational perspective is the *form* of the relation, the kind of structure linking two or more objects. In this respect, genealogical relations *as such* are not relevant, insofar as they would generate very broad classes: for instance, all humans and mice share the same formal genealogy (they have all two parents, each of which has two parents.). Accordingly, more restrictive interpretations of the relational properties of organisms are adopted, as we discuss below, mainly focusing on their observable functioning and organization. Moreover, as mentioned, the relational epistemology holds that the mathematical form of the relations is supposed to remain stable in time. Relational identity requires, therefore, the stability of the relevant properties, when considering both the synchronic and diachronic identity of a group of organisms. The contrast with the genealogical conception, which characterizes organisms' identity without relying on stable properties, is sharp.

In biology, we distinguish two versions of the relational epistemology and the resulting conception of identity. A first version, adopted in particular by biophysics and systems biology, consists of studying biological organisms by using conceptual and mathematical tools common to other natural sciences, as physics or chemistry. While it relies on well-established and operational tools, this “biophysical” version tends to look at biological organisms as physicochemical systems and, therefore, to emphasize common aspects while neglecting specifically biological ones. The resulting conception of biological identity applies to those aspects, and their relations, which are captured by the models. Different organisms are synchronic instances of the same object insofar as they possess the same aspects and relations captured by the model, and they maintain their identity diachronically if they conserve them in time.

The main strength of the biophysical conception of identity is that, in contrast with the genealogical one, it makes explicit the conditions of validity of experimental results. Generalizability and reproducibility of results hold for all organisms belonging to the same identity class, insofar as they possess the aspects and relations made explicit by the model or description. At the same time, this definition carries a crucial weakness: it considers *exclusively* these aspects. Biophysical identity applies only by abstracting from any other aspect or property of organisms not included in the description. By “abstracting,” we mean that all other aspects of the organisms are supposed to be negligible for the compliance with the model.

The problem with this abstraction move is two-fold. On the one hand, it implies dealing with organisms not as wholes, but as circumscribed sub-systems. In fact, biophysical models used in biology often apply also to abiotic phenomena (Douady and Couder, 1996; Fleury, 2009). If relational identity is built upon such a restricted characterization of the organism, one can wonder whether it constitutes a relevant criterion of organisms' identity given that, in a sense, it neglects most of the organism. On the other hand,—and crucially—the abstraction does not work most of the time. Experimental biologists and modelers are usually not able to abstract from all other aspects, which prove to be not negligible and generate observable differences between organisms (Isaacs, 1986; Mogil et al., 1999; Festing, 2014). As a result, individual organisms typically exhibit significant variability with respect to a particular model, and observations contradict their purported relational identity. Therefore, while its domain of validity is explicit, biophysical identity is seldom valid.

The second version of the relational epistemology, which we label “organizational,” places a heavier emphasis on the distinct features of biological organisms. Its central assumption is that organisms are natural systems endowed with a distinctive organization. In particular, biologists can analyze organisms (be them unicellular or multicellular) as constituted of parts that depend on each other for their continued existence: biological “organization” refers specifically to such a mutual dependence among parts. Initially advocated by theoretical biologists like Nicolas Rashevsky (1954) and Robert Rosen (1991), the organizational epistemology is in a way “more relational” than the biophysical one because it focuses on the fact that organisms realize a distinctive regime of relations between their parts. Classical accounts of the organizational framework are Varela and Maturana's autopoiesis (Varela et al., 1974), Rosen's (*M, R*) systems (Rosen, 1991), and Kauffman's autocatalytic sets (Kauffman, 1993).

**Figure 1:**
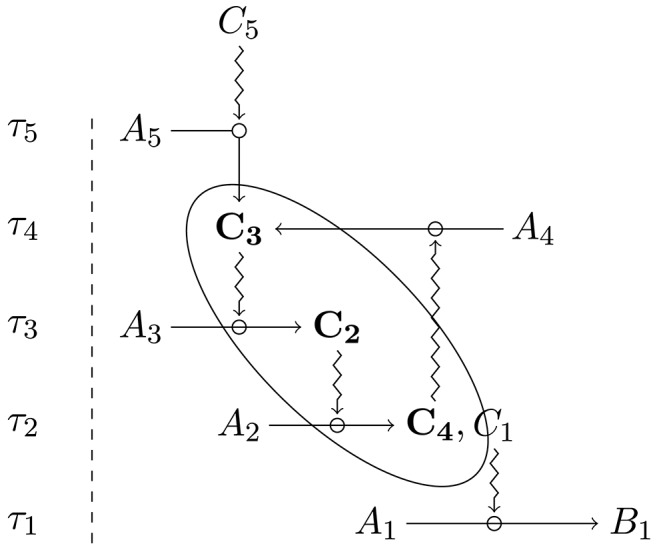
In this diagram, *C*_1_, *C*_2_, *C*_3_, *C*_4_, and *C*_5_ play, ex hypothesis, the role of constraint at τ_1_, τ_2_, τ_3_, τ_4_, and τ_5_, respectively. Furthermore, *C*_1_, *C*_2_, *C*_3_, and *C*_4_ are dependent constraints, while *C*_2_, *C*_3_, *C*_4_, and *C*_5_ are generative constraints. The subset of constraints that are both generative and dependent is then (*C*_2_, *C*_3_, *C*_4_). The organization constituted by *C*_2_, *C*_3_, and *C*_4_ realizes closure (reproduced from Montévil and Mossio, 2015, with permission from Elsevier).

Let us describe in some detail the central tenets of this organizational framework, by relying on some recent theoretical developments (see also Montévil and Mossio, 2015; Moreno and Mossio, 2015; Kauffman, 2019, for recent discussions). One of the central aims of the organizational perspective is to provide a fine-grained characterization of the mutual dependence between an organism's parts, which in turn brings about the idea of circularity. Biological organisms are understood as natural systems realizing a dual causal regime. On the one hand, they are thermodynamically *open* systems: they are traversed by a flow of energy and matter that enables them to maintain themselves over time in conformity with the second principle of thermodynamic. On the other hand, biological organisms control the thermodynamic flow through the action of structures that, at specific time scales, exert constraints on the ongoing processes and transformations. In particular, organisms are constituted by a set of constraints that (1) are generative—they canalize target processes in such a way to maintain the conditions of existence of other constraints and (2) are dependent—their existence relies on the action of other constraints (see Figure 1).

The set of constitutive constraints that are both generative and dependent realize mutual dependence, which is usually referred to as *closure*. One of the conceptual strengths of the organizational perspective is that it provides an account for the concept of biological function, defined as the effect produced by a constraint subject to closure (Mossio et al., 2009; Nunes-Neto et al., 2014). By realizing closure of constraints, the organism maintains itself. In turn, the otherwise general idea of “biological organization” is defined as closure: for an organism to be organized means realizing closure of constraints (Montévil and Mossio, 2015, for details).

Organizational closure provides a specific interpretation of the circularity at work in biological organisms (Mossio and Bich, 2014). Importantly, the closure principle provides theoretical guidance to explain the relative stability of biological organisms. Functional constraints exhibit conservation at the time scale at which they act on processes: as claimed elsewhere (Montévil and Mossio, 2015), it is precisely their local conservation that endows them with the capacity to control the thermodynamic flow. At longer time scales, however, constraints undergo degradation and must be repaired or replaced: this is where organizational closure steps in and contributes to explain how organisms as wholes stabilize themselves over time.

With this brief characterization in hand, let us examine how the organizational framework deals with organisms' identity. As for any conception of identity, different interpretations of the organizational one can be adopted. The most restrictive relative interpretation seems to be that different organisms are instances of the same object insofar as they share the very same functional organization, i.e., if they realize (at some given stage of their lifetime) the closure of the *same* constraints. At the opposite end, the most inclusive definition would state that different individual organisms are identical if they merely realize closure, whatever specific set of functions is involved.

As a matter of fact, Difrisco and Mossio (In press) have recently argued that the most inclusive interpretation of organizational identity is well suited to account for organism diachronic identity. A given organism remains the same, despite any kind of change that it can undergo (especially during development), if it realizes a continuous succession of regimes of closure, such that each regime depends on some functional constraints exerted by a previous regime. The connection between different regimes of closure that grounds diachronic identity is what DiFrisco and Mossio call *organizational continuity*. For the purposes of this paper, which focuses on the conception of organisms' identity relevant for modeling and experimental purposes, the most inclusive interpretation of organizational identity looks inadequate. By hypothesis in the organizational perspective, all organisms realize closure; therefore, the general criterion of closure would include a massive number of very diverse organisms, which would prevent generalizations and reproducibility in most cases. A more restrictive interpretation, warranting some functional similarity between organisms, seems to be required.

Let us now consider the most inclusive interpretation, according to which organisms are identical if they realize the closure of the very same constraints. We consider here^3^ that two or more constraints are the same in organizational terms if they perform the same function, which means that they constrain the same kind of processes by relying on the same kind of mathematical or geometrical structure. For instance, two constraints are instances of the same vascular system if the same topological structure of vessels constrains the transport of oxygen and nutrients to cells, and of wastes afar from them. The emphasis here is on the qualitative, functional identity between constraints, while limited quantitative differences are negligible. In contrast, quantitative differences between functionally identical constraints may be relevant when comparing whole organizations, insofar as they can lead to a qualitative difference in some *other* constraints and, therefore, in the way overall closure is realized^4^.

To the extent that organizational closure is a distinctive feature of biological organisms, this relational conception of organism identity seems to be more suitable because it avoids the first possible drawback of biophysical ones, i.e., the fact of leaving aside specifically biological aspects. Indeed, identity grounded on closure naturally considers organisms as whole entities. As for the biophysical conception, the organizational one makes explicit its domain of experimental validity. To be the same, different organisms must share the same organization. In contrast to the biophysical definition, however, an explicit description or model of the *whole* functional organization of an organism appears to be out of reach for the scientific inquiry. As a result, the criterion is not directly applicable. One could argue that it constitutes the “horizon” of a well-grounded definition of biological identity or, on the opposite, that a complete description of an organism might also prove impossible to obtain in principle.

A possible solution to the problem would be to establish descriptions and models of *partial* closure, and take them as criteria of identity. By “partial closure,” we mean a closure among a subset of all functional constraints constituting a given organism. For instance, a given model can specifically focus on the reciprocal dependencies between constraints of the respiratory and vascular systems, under the hypothesis that these are critical for the cohesion of the whole organization. Accordingly, we distinguish models of *partial* closure from *local* biophysical models: while the former describe parts of an organism that do realize closure, the latter do not.

One may object that such a solution would also face the problem of abstracting most of an organism's organization, just as the biophysical one. With no theoretical guidance, partial models would neglect aspects that might actually make a difference and induce variability between supposedly identical organisms. The objection is undoubtedly correct. Yet, we submit that the organizational framework has better prospects than the biophysical one for selecting relevant aspects of an organism within an adequate theoretical framework. The reason is that even partial organizational models are nevertheless models of closure (while biophysical ones are not) and therefore designed to account for the reciprocal stabilization of functional constraints within whole organisms. As a result, they can better determine the occurrence and impact of variations affecting organisms and the extent to which such variations could alter their identity.

## 3. An Hybrid and Bounded Conception of Organisms Identity

The upshot of the previous section is that genealogical and relational conceptions of organisms' identity have complementary strengths and weaknesses. In what follows, we advocate their integration into a hybrid conception that, we hold, is better suited for taking up the challenge of organisms' complexity.

The connection with a fixed past allows the genealogical conception to define organisms' identity in a way that accommodates biological variations. However, genealogical identity does not refer to any observable property of organisms, which leaves unspecified to what extent experimental generalizations are legitimate. In sharp contrast, relational identity refers to the observable properties of organisms, which provide specific conditions for scientific generalization and reproducibility. Yet, relational identity faces the problem of abstraction with regards to most of an organism's organization, with the result that it seldom proves valid.

The reason why relational identity fails to apply to organisms easily is not only that a complete description of their organization is not accessible. Even if a complete description of an organization were available, we submit that the corresponding biological organisms would undergo *unpredictable* variations. Biological variation in such a “strong” sense is not merely quantitative; it corresponds to the appearance of structures, processes, couplings, and functions that are fundamentally *new* (Longo, 2018; Kauffman, 2019; Montévil, 2019b). Elsewhere, we have argued that the appearance of unpredictable variation in biological organisms should be a fundamental principle of biology—the *principle of variation* (Montévil et al., 2016)—which governs biological phenomena together with the principle of closure.

**Figure 2:**
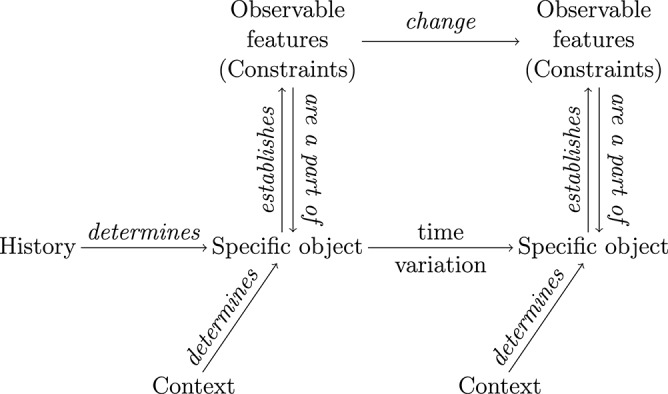
Integration of genealogical and relational descriptions (reproduced from Montévil, 2019a, with permission from Springer). Relational concepts, constraints here, are insufficient to define specific objects: they are fundamentally historical. They nevertheless possess relational properties, constraints, that are valid for some time, and can change over time. This schema has been designed for biological organisms and is a starting point to integrate genealogical and relational identities.

In this situation, we submit that an adequate conception of organisms' identity requires integrating genealogical and relational (organizational) strategies, as Figure 2 illustrates. Organisms are *specific* objects, which means that each of them can possess specific features that make it qualitatively different from other organisms to an extent. Organisms are specific objects because they are the result of a history of variations, and they continue to undergo further variations over time. Yet, in any given experimental situation, a group of organisms can also be shown to share some *generic* (i.e., common) aspects, typically constraints, captured by a relational description and supporting generalization. Over time, however, biological variations may involve a change of these constraints even in controlled laboratory strains. Such changes would make the identity grounded on the hybrid conception invalid. Let us discuss in some detail the central tenets of the conception we advocate.

### 3.1. Conceptual Tenets

Physicists understand the changes taking place in a given phenomenon by variables connected by invariant relations, expressed as equations. By contrast, following the principle of variation, we submit that there is no invariant mathematical structure (as equations) underlying the behavior and dynamics of organisms.

A central epistemological implication is that we have to understand the relative stability of biological phenomena without overarching invariants. As mentioned in the previous section, organizational closure plays precisely this epistemological role at the individual scale, by contributing to explain how functional constraints stabilize each other through their reciprocal relations and interactions. As recently argued (Mossio and Pontarotti, 2019), closure can also explain the stability of functional constraints across generations by providing an organizational understanding of biological heredity. Natural selection plays a similar role at the evolutionary scale, in that it excludes some trait variants and, thus, explains the stability of other variants, as adaptations (Lecointre, 2018). To the extent that both closure and natural selection are the basis of philosophical accounts of the concept of “biological function,” the ascription of functions is typically understood as a way to explain the stability of function bearers at the individual and evolutionary scale (Montévil, in press).

How should organism identity be characterized in this theoretical framework? We propose six main tenets. First, organism identity requires elaborating a generic description of organizational closure, which is supposed to apply to a group of individual organisms. Such a description aims to capture not only the relations between functional parts of an individual organism but also, and crucially, its interactions with the environment as an agent (Barandiaran and Moreno, 2008), as well as with other organisms (Hernández and Vecchi, 2019).

Second, organizational descriptions are necessarily partial, despite their possible complexity. This limitation implies that many aspects are neglected, be they other functional parts, or aspects of the environment, or other organisms. In section 2.2, we referred to this implication as the *abstraction* made by relational models. The ineluctable abstraction of the organizational description means that the neglected aspects are also uncontrolled and might, therefore, hide relevant differences between the individual organisms. Because of the complexity of biological organisms discussed in the Introduction, such differences do exist most of the time, and prevents using explicit organizational descriptions as a sufficient criterion to build identity classes.

Third, the genealogical strategy steps in and provides a procedure for dealing with the aspects that the organizational framework does not make explicit. The procedure considers as candidates for membership to an identity class those organisms which share the same past. Often, in experimental biology, organisms have a controlled, *recent* common ancestor (even though other aspects of their past may also be controlled, see Montévil, 2019a). Under the implicit assumption that the closer organisms are to a common ancestor, the more they tend to share generic aspects, such a procedure provides indirect control on those aspects neglected by the organizational description^5^. These neglected aspects include not only parts of organisms but also the environment of successive generations leading to them, as well as other features that may be interpreted as belonging either to the former or the to latter, such as the microbiome of mammals. Since biologists cannot completely describe organisms in relational terms, they use the genealogical strategy that *complements* the organizational description.

To illustrate how the genealogical strategy fills in the gaps of the organizational one, let us focus on the treatment of specific functional constraints. A constraint is a relational concept, defined by its mathematical structure and its link with the constrained process (Montévil and Mossio, 2015). However, the isolated description of a constraint within an organism is not exhaustive, insofar as it omits other constraints that may contribute to its stabilization (be it at a higher level or the same level of organization) or may constitute it at a lower level. For example, physicists can analyze the camera eyes of mammals and cephalopods with a single optical model; yet, the details concerning the nerve position, vasculature, molecules are very different, and so are the possible relations with other functional constraints, as well as variants, pathological or not. That is why the genealogical concept of *homology* enters the picture naturally. Homologous constraints tend to be constituted by (and articulated with) other constraints displaying a higher degree of similarity, in comparison to the situation of analogous constraints. Actually, the genealogical connection that matters here can be more specific than the one captured by the concept of homology alone, insofar as relevant constraints would come from specific genealogical groups, such as specific species or strains. Such genealogical control is a critical asset when dealing with organizations that have no complete relational description. As a result, the historical characterization of constraints identity complements their relational description. Functional constraints are the same when they have the same historical origin *and* share the same relational properties.

Fourth, the organizational conception focuses on constraints closure, which contributes to explain how biological organisms can maintain themselves over time by constraining the thermodynamic flow. In particular, closure brings about an inherent tendency of organisms to stabilize existing functional constraints by removing many variations and by regenerating them in a fundamentally unaltered form. Such a tendency to conservation (what we have previously labeled “organizational inertia” in Mossio et al., 2016, section 5.1) would notably apply in those situations in which variations are circumscribed and do not affect the constraints in charge of regenerating the one (or set) being affected. In these situations, organizational closure tends to restore the initial constraints. In other words, organization imposes theoretical conditions on the kind of variation that is likely to be preserved^6^. Moreover, variations need to be significant for the description in terms of closure of constraints. The appearance of such functional novelties typically takes time. It requires the emergence of a specific constraint and its integration to the organization. Such an outcome is not the result of generic randomness; it requires finding a new specific functional organization by constituting and exploring new configurations (Montévil, 2019b).

Fifth, the tendency to conservation emphasized by the organizational framework *provides theoretical support* for the hypothesis according to which genealogical proximity tends to go with organizational proximity. Because of this tendency, together with the fact that the emergence of functional novelties takes time and natural selection, the closer genealogically organisms are, the less they tend to differ. It might be argued that organizational novelties may sometimes be significant over a relatively short period, for example, within one generation, because of phenotypic plasticity (West-Eberhard, 2003). The point is certainly right; still, it seems correct to point out that these changes are quantitatively limited in comparison to the bulk complexity of biological organizations. The overall result integrates genealogical and relational conceptions of identity: the former fills in gaps of the latter, which in turn justifies some implicit assumptions of the former.

Sixth, the integration between genealogical and relational conceptions leads us to advocate a *hybrid* conception of organism identity. Individual organisms are members of the same identity class if they have a high degree of genealogical proximity *and* they share a distinctive, specific regime of organizational closure. Let us assume, for instance, that biologists want to study the flight of bats. Two organisms are experimentally identical bats if they descend from a close common ancestor *and* they share a specific set of organized, functional constraints as those involving flight, which include (among other things) the anatomy of their wings. Biologists would also exclude bats with congenital abnormalities affecting wings and other variations impacting the relevant properties involved in bat flight. We submit that such a hybrid definition of organism identity keeps the benefits of both genealogical and relational conceptions while avoiding—or at least mitigating—some of their central drawbacks.

Yet, the hybrid nature of the definition is not the end of the story. Indeed, our theoretical framework relies on the principle of variation, according to which individual organisms do undergo variation over time. The main implication here is that, even though an individual organism satisfies the hybrid conception at a given moment, there is no guarantee that it will do so as time passes. Consequently, although a population of organisms shares the same hybrid identity during several generations, sooner or later, some of these organisms will undergo variations that will contravene their membership to that identity class. As a result, our conception of organism identity is not only hybrid but also *bounded* in time.

### 3.2. Toward a Theoretical Characterization

The conceptual framework outlined above would gain clarity if it were expressed by an adequate formal language, which, to our knowledge, is currently lacking. Let us take some preliminary steps in this direction.

We first introduce a new symbol, χ, which represents the *historical aspects* of organism identity. χ relies on a genealogical connection with an ancestor, or more generally with the past, and complements relational descriptions of organisms. Accordingly, it includes all those aspects of identity which are not made explicit by the relational part of any given description. In conformity to the features of genealogical identity, χ accommodates past variations and contexts that have shaped the present (group of) organism(s) in evolutionary and ontogenetic time. As such, theoretical and relational invariants do not define χ, although it might include stable relations that have remained implicit or neglected (voluntarily or not, see also below) in the relational description.

In any characterization or model complying with the hybrid conception of organisms' identity, χ realizes organizational closure in combination with the constraints explicitly represented in relational terms. The overall characterization does not make the closure entirely explicit, precisely because it contains χ. A group of organisms that meet the hybrid model—and would, therefore, share the *same* explicit relational description and the *same* χ—would share the same identity, even though they could nevertheless hide some differences, because of the very nature of χ. At the same time, χ can also contain some implicit stable relations due to the organizational tendency to conservation, as mentioned in the fourth tenet. Genealogical strategies of symmetrization exploit this tendency and provide some control over χ (typically, by selecting different organisms having a close common ancestor). Together, the explicit relational description of the constraints and χ generate an identity class adequate for experimental work.

Since there is no theoretical invariant specified by χ, its status is fundamentally different from that of a variable, as used in physics. Variables are defined through formal relations, while χ refers to a genealogical connection with a specific object, a particular. As a result, it is ultimately a symbol in the etymological sense of the word, bridging the formal description and the part of the world under study.

How is χ formally integrated into an organizational model or diagram? The general idea is to represent χ as a *sui generis* constraint subject to organizational closure. As such, χ is understood as being both dependent and generative for some other constraints of the diagram. Yet, the specific nature of χ implies that its relations with the rest of the system have a special meaning. To a first approximation, we submit that the integration of χ to organizational closure, rather than representing actual relational knowledge, consists of a *background assumption* that requires a conceptual justification and a formal representation. Let us discuss these issues in some details.

**Figure 3:**
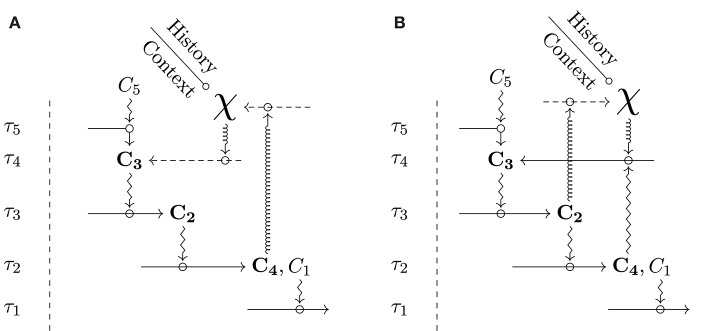
Integration of a historical symbol and organizational closure. Since χ and the relational constraints have a different epistemological nature, we use different arrows for constraints and processes related to χ. *Zigzag arrows* are relational constraints; *straight arrows* are processes; *spring arrows* represent constraining effects that relate to χ and are therefore not entirely relational; *dashed arrows* indicate hypothetical processes constrained by spring arrows. Constraints are defined in relational terms while χ is defined genealogically, by reference to the past. In **(A)**, there is a global closure that involves χ, while **(B)** includes an additional partial closure of constraint in relational terms.

Figure 3 shows two kinds of diagrams that realize organizational closure by integrating χ. Figure 3A provides the most general version, in which there is only one global closed path of constraint dependencies, which includes χ. In turn, Figure 3B describes a situation in which, in addition to the global closure, a *partial* closure is realized among the constraints, independently from χ. Because of the specific nature of χ, the global closure that includes it has a hypothetical status and does not count as a legitimate *model* of an organism. Hence, the kind of diagram depicted in Figure 3A requires a justification within an organizational framework, typically by exhibiting empirically relevant examples that satisfy the diagram and *also* realize partial closure. In a nutshell, we can justify Figure 3A if it has concrete instances like in Figure 3B. With this justification, biologists can legitimately use a diagram with no partial closure, insofar as it is not always necessary to explicitly represent the latter in a model, and some aspects of organizational knowledge can be left implicit^7^. With these clarifications in hand, we can use diagrams of both Figures 3A,B to build hybrid identity classes for groups of organisms in the context of modeling and experimental practices. The more constraints are included, the more the interpretation of identity (and the resulting classes) is restrictive, and the more stringent empirical checking has to be. Similarly, the more strict the tentative experimental, genealogical control exerted on χ is, the more restrictive the class is.

Diagrams integrating χ to organizational closure raise the question of the connection between χ and the explicit relational part. Depending on what the modeler knows and ignores about the organisms, the diagram has a different meaning and form, in particular with regards to the dependencies between χ and other constraints. Besides, if the diagram does contain a partial closure, specific organizational patterns become visible, and further general challenges arise. For instance, as one can see in Figure 3B, the coexistence between global and partial closure seems possible only if χ depends on, and maintains, at least one constraint (not necessarily the same) that is also part of the partial closure. This situation implies—among other things—that at least one constraint in the diagram must perform multiple functions. Understanding how this organizational pattern can be realized (or how another pattern can produce the junction) is a typical example of a general scientific question raised by the inclusion of χ to closure.

When considering the relations between χ and the constraints in a diagram, we can distinguish several cases. Without trying to be exhaustive, let us mention a few significant ones. It is worth noting that these cases are not supposed to be mutually exclusive: the very same χ in the same diagram can carry aspects that are relevant for several of these cases.

The first case is a generalization of the situation that we discussed earlier for Figure 3A. In a given diagram and situation $D^{0}$, χ might refer to organisms where other aspects could be made explicit in relational terms in a different diagram $D^{1}$. So to speak, there is some knowledge that can be “unpacked,” if required. This operation can imply a transition from a model with no partial closure to a model with partial closure (as discussed above) or from a model with partial closure to a model with an enriched partial closure. The central idea, here, is that part of the situation described by the initial χ can be described by a set of organizational features that are, at least to some extent, known to be generic, i.e., common to several organisms sharing the initial hybrid identity. Accordingly, these features could be explicitly integrated into a new model determining a more restrictive hybrid identity formally, $D^{1}$. The latter may exclude some concrete organisms which were previously included by $D^{0}$. The choice between $D^{0}$ and $D^{1}$ ultimately depends on the specific epistemological, experimental, and modeling objectives pursued. For example, the constraints involved in cellular respiration are mostly generic in the sense of being relatively common to, say, all mammals and, therefore, could be left implicit in models focusing on other aspects unless the model is explicitly aiming at providing a relational characterization of oxygen transport. Formally, there are two ways to link the initial diagram $D^{0}$ and the new one $D^{1}$. If we use $D^{1}$ instead of $D^{0}$, the diagram change corresponds to a change of identity. Alternatively, one may keep the initial identity and justify the articulation between the constraints and χ by the subclass $D^{1}$ describing a partial closure that includes the constraints explicit in $D^{0}$. In this case, $D^{0}$ is complemented by a special case, $D^{1}$, that justifies the articulation between χ and constraints in $D^{0}$. This justification does not guarantee that the constraints under study are always functional in $D^{0}$; however, it guarantees that they are in some cases. We can thus see $D^{1}$ as an “organizational type” of $D^{0}$, and write this concept as $D^{0}\left[D^{1}\right]$. In a given situation, when the constraints involved are largely conserved, we can argue that $D^{1}$ is representative of most cases, then other situations will be exceptions.

In the second case, we postulate that some aspects of χ are equivalent to aspects explicitly described in relational terms. The underlying hypothesis is that a constraint may have a single generic effect on a class of processes having different roles in the organizational diagrams. For example, cell membranes constrain the diffusion of a broad class of molecules similarly, or ribosomes constrain the translation of most RNAm similarly. In particular, a constraint can act in the same, generic manner on a process contributing to the partial closure *and* have an effect on χ in the global closure. Figure 3B somehow captures this situation: constraint *C*_2_ acts on the process maintaining *C*_4_ and on a process acting on χ. The critical point is that the way such a constraint acts does not require us to specify the process constrained; instead, this process just needs to be in the target class, and we need to assume that maintaining χ requires such processes — a valid assumption for the membrane and the ribosomes. Let us take another biological example. In a mammal, the constraints involved in oxygen transport (among others, and roughly speaking, those of the vascular systems and the lungs) lead to oxygen distribution to all organism's cells. Cells depending on oxygen distribution include those of the vascular systems and the lungs themselves, which allows drawing a partial closure among them. Moreover, we can safely claim that almost all other cells in the organisms depend on these constraints. This claim justifies the assumption that the constraints are also involved in the global closure^8^. The way this dependence is materialized is, however, extremely diverse because oxygen, and respiration, enable cells and organisms to perform all kinds of processes: there is a generic dependence on respiration. Under the assumption that the constraints involved in respiration are generic, a theoretical connection can, therefore, be established between χ and the relational description (which can include or not an explicit partial closure) without needing an explicit relational description of the purportedly relevant aspects of χ.

The third case refers to a situation in which, although χ could be “unpacked,” as discussed above, the resulting organizational model would be extremely specific, and therefore unfit to sustain generalization and reproducibility. In other words, the transition from an initial diagram $D^{0}$ to a new, more complex one would tend to make specific relational aspects explicit rather than generic ones. As a result, the identity class would become extremely restrictive, and only a small subgroup of organisms (if not just one) would meet the criteria. For example, the regulatory effects of thyroid hormones can be radically diverse, as shown by examples like frog metamorphosis or mammal hibernation, among many others. Trying to elaborate an organizational model which would include the various effects of these hormones and, at the same time, would apply to a broad group of organisms, would presumably be a dead-end initiative. In this case, χ accommodates a diversity coming from past novelties that is irreducible to an organizational model that would aim to generate an inclusive identity class. Let us call $D^{0}$ the initial diagram and $D^{1,1}$, $D^{1,2}$, $D^{1,3}$, ., other more specific diagrams where a relational closure is explicit^9^. Then, like in the previous case, one may choose to work with a different object, having a different identity, say $D^{1,1}$. Again like before, one may instead consider the $D^{1,i}$ as organizational types of $D^{0}$, written $D^{0}\left[D^{1,1},D^{1,2},D^{1,3},...\right]$. Then, we make explicit that the constraints of $D^{0}$ may be functional in a diversity of ways. The fact that organizational models $D^{1,i}$ do not possess an acceptable degree of generality does not imply that they have no epistemological role. They increase biological knowledge by showing that specific constraints can have functions in a given class, even though in a diversity of ways.

The fourth and last case that we discuss here concerns the situation in which χ includes intrinsically diachronic constraints. As such, these constraints may involve novelties that have not appeared yet and whose nature may be unprestatable (Longo et al., 2012; Montévil, 2019b). Consequently, these constraints are only *potentially* functional in relational terms, and their position in the organizational diagram can be assessed only ex-post. One notable example is the “propulsive constraints” described by Miquel and Hwang (2016) following previous analyses by Canguilhem (1972). Propulsive constraints promote the appearance of novelties that are unpredictable and even unprestatable. For example, the “mutator system” is a regulation of the mutation rate of DNA exerted by specific molecular constraints. Bacteria under stress can reduce mutation corrections, which increases mutation rates and allows exploring new organizational possibilities (Miquel and Hwang, 2016). The emerging capacities and constraints can be functional, but the mutator system *itself* , as well as other relational properties of the initial organization, do not specify the features of these new constraints. As a result, the mutator system cannot be located into an organizational diagram, insofar as its functional contribution is unknown a priori. As for the previous case, we can use organizational types to justify that the constraints of the mutator system are functional $D_{t_{1}}^{0}\left[D_{t_{2}}^{1,1},D_{t_{2}}^{1,2},...\right]$, with *t*_1_ < *t*_2_. However, there are two critical differences with the previous case. First, the organizational types are not at the same time point. Second, it is not possible to avoid using types and only study $D_{t_{2}}^{1,1}$ because the latter does not make the function of the propulsive constraints explicit. The fact that the mutator system cannot be included in a general organizational model does not imply that relational descriptions are not useful. In all those cases in which the increased rate of mutations triggers the emergence of functional changes in organisms, specific organizational models can account for the new functional role, and therefore justify the function of the mutator constraints.

The integration of χ within organizational models covers a variety of situations. Following the specific scientific objectives and depending on the available knowledge, the relational part of the diagram can be more or less detailed, and generate more or less restrictive hybrid classes of identity (together with the genealogical control on χ). Yet, it is worth underscoring that, as we discussed in section 2.2, we maintain that an organizational description is never complete (be it for contingent or principled reasons), which means that whatever model of an organism does include χ. Organisms' historical identity possesses irreducibility that cannot be captured by any given organizational model.

**Figure 4:**
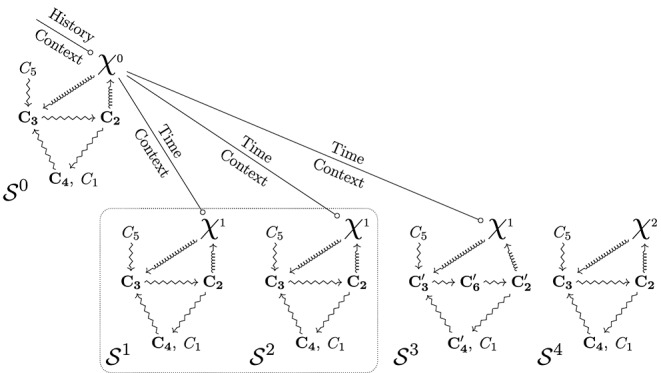
Theoretical representation of a typical experiment. **(Top)** $S^{0}$ is a specimen that is a common ancestor to the organisms studied in the experiment. This specimen may be identified, or its existence may be theoretical, in which case another particular serves as a reference, like in systematics. Accordingly, the existence of the specific constraints, *C*_*i*_, for this specimen may be an empirical observation or a hypothesis. **(Bottom)** several specimens are generated, possibly after multiple generations. Their genealogical identity (including their context) is considered equivalent; therefore, we use a single symbol, χ^1^. $S^{1}$ and $S^{2}$ have the same hybrid identity because both their genealogical *and* relational components coincide. Of course, if we were to investigate other aspects accommodated by χ^1^, we would find qualitative differences between these two specimens: χ is defined genealogically and is compatible with such variations. In the case of specimen $S^{3}$, the variations lead to a change in the constraints described; here, *C*_2_ becomes $C_{2}^{\prime }$, and there is a new constraint $C_{6}^{\prime }$. As a result, this specimen escapes the relational part of the hybrid identity class of $S^{1}$ and $S^{2}$. Note that, for $S^{3}$, the symbol χ^1^ remains the same as for $S^{1}$ and $S^{2}$ because the genealogical identity remains the same. If a biologist wants to investigate the nature of the variations leading to the change of constraints observed, then other constraints have to be made explicit. This operation would lead to a different definition of the class of $S^{3}$. Last, $S^{4}$ possesses a different χ. The corresponding constraints may be analogous, or χ^2^ may correspond to a different strain or species where the constraints described are homologies. Consequently, it does not belong to the same identity class of $S^{1}$ and $S^{2}$, but the reason is contrary than for $S^{3}$.

By characterizing the identity of organisms for modeling and experimental practices, organizational diagrams integrating χ can also represent a typical experiment. Before concluding this section, let us have a brief look at this application of the framework (Figure 4). In a typical experiment, several organisms ($S^{1}$, $S^{2}$, $S^{3}$, and $S^{4}$) are candidates as a support to enquiry on the properties of some target relational capacities and features (represented in Figure 4 as the constraints *C*_1_-*C*_5_). Each organism is characterized by a diagram including both the constraints under scrutiny and the symbol χ. Being the offspring of the same common ancestor, specimens $S^{1}$, $S^{2}$, $S^{3}$ share the same χ (i.e., χ^1^) and are therefore genealogically identical. Moreover, $S^{1}$ and $S^{2}$ also share the same relational description of the target functional constraints. Consequently, $S^{1}$ and $S^{2}$ share the same hybrid identity as defined by the model, and they can be tentatively defined as two instances of the same experimental object. In contrast, specimen $S^{3}$ does not share the same identity because it exhibits significant variations in its relational description: despite having the same χ^1^ than its relatives, its *relational* difference breaks the criteria for membership in this specific hybrid identity class. Specimen $S^{4}$, in turn, shares the same relational description than $S^{1}$ and $S^{2}$ with respect to the target constraints, but it does not share the same *genealogical* connection with the past. This difference excludes it from the same identity class (for opposite reasons when compared to $S^{3}$). Although this case may seem paradoxical since it looks identical in relational terms, its exclusion from the identity class is theoretically justified precisely because historical identity is taken into account: accordingly, a different χ may, and will, carry hidden differences.

Overall, the diagrams represented in Figures 3, 4 build hybrid identity classes of organisms. In a nutshell, a hybrid identity class integrates genealogical aspects represented by χ and relational ones represented by all the constraints. Organisms may violate the relational description in time, which is why the hybrid identity is also bounded. In some cases, as mentioned, the proper justification of such diagrams requires the use of organizational types, which are more restrictive classes than the initial one.

## 4. Conclusions

Biological organisms are a very peculiar kind of natural systems. They are familiar to us and, at the same time, resistant to a comprehensive scientific understanding. As claimed in the Introduction, they are complex objects.

The characterization of organisms' identity faces their complexity. It is a notoriously difficult task to tell whether a group of organisms that look similar at first sight does not hide substantial differences, which may be revealed after in-depth scrutiny. Similarly, it is difficult to make explicit the conditions at which it is legitimate to claim that an organism remains the same over time. Despite these challenges, a workable notion of organisms' identity is required, because of its pivotal role in grounding generalization and reproducibility in science.

In this paper, we have discussed the strengths and weaknesses of two broad conceptions on identity. The genealogical conception builds identity classes by reference to the past, especially by linking individual organisms to a common ancestor. Experimental biologists routinely use this strategy to work on hypothetically equivalent organisms. While it tends to work, genealogical identity does not provide its conditions of validity for experimental purposes. The relational conception, in turn, defines identity by referring to a set of relations possessed by individual organisms. While its conditions of validity are explicit, it faces the widespread problem of biological variability.

To overcome this situation, we have put forward a hybrid conception of organisms' identity. We have argued that the identity of biological organisms should be construed by integrating both genealogical and relational conceptions. In short, we suggest that individual organisms belong to the same identity class when they share the same specific organization of functional constraints *and* they are the offspring of the same close common ancestor. The two poles of the definition are complementary, in the sense that they provide mutual support and contribute to filling in their reciprocal gaps. The genealogical conception provides an operational procedure to subsume whole organisms to the same identity class, even though no complete relational description is available; in turn, the relational conception—in particular in its organizational version, that we adopt—provides a theoretical justification of the implicit hypotheses underlying the genealogical one. In the last section, we have provided a preliminary formal representation of biological hybrid identity, by introducing a symbol, χ, that accommodates the contribution of the genealogical conception of identity, within an organizational description of an organism. The formal representation of history within a relational diagram is a stimulating challenge that future studies should take up. Our discussion suggested that χ allows describing different possible connections between the historical and organizational dimensions of organisms, as well as their implications for experimental and modeling practices.

Even though the hybrid definition of identity was deemed to be useful and fecund in the biological domain, we have also underscored that the validity of identity classes cannot be but limited in time. Because of their inherent tendency to vary, individual organisms that meet the criteria of an identity class at some moment may contravene these criteria as time passes, and their offspring will presumably do the same after some generations. Therefore, organisms' identity is not only *hybrid* but also *bounded*: both aspects draw a fundamental difference between biology and other natural sciences.

## Author Contributions

All authors listed have made a substantial, direct and intellectual contribution to the work, and approved it for publication.

### Conflict of Interest

The authors declare that the research was conducted in the absence of any commercial or financial relationships that could be construed as a potential conflict of interest.

## Footnotes

^1^By contrast, properties that do not stem from relations are arbitrary. For example, in classical mechanics, both stillness and uniform movement correspond to no force, thus ultimately to the same situation. In Galilean relativity, the difference between the two situations stems only from the arbitrary choice of a reference frame; choosing a different reference frame can transform the stillness of an object into uniform motion.

^2^Current debates in physics concern the alternative between the possible use of absolute concepts (such as the absolute time of Newton) or the adoption of a *purely* relational epistemology. However, both positions acknowledge that physics relies *mostly* on a relational conception (Huggett and Hoefer, 2018).

^3^A full-fledged discussion of constraints identity goes beyond the scope of this paper. As detailed in Montévil and Mossio (2015), the formal definition of constraints appeals to conserved properties, which enable them to produce a causal effect on a target process. A precise characterization of their identity should, therefore, take into account these aspects.

^4^Let us mention that the issue is complex since mathematical descriptions, especially equations, precisely subsume a diversity of situations under the umbrella of a single mathematical frame. As a result, different views coexist. Two systems may be considered different on quantitative bases, either by their states (different positions) or their parameters (different mass). On the opposite, they may also be different if the overall equation representing them is different. Last, there are situations in between. For example, physical morphogenesis or bifurcation are situations where a change of state corresponds to a qualitative change of the trajectory or structure of the object.

^5^The idea behind genealogical proximity can be understood from a more general perspective in terms of *symmetrizations* (Montévil, 2019a). Symmetrization refers to all methods adopted to handle the historicity of living organisms, so as to make them tentatively identical, and to enable biologists to perform reproducible experiments. In addition to genealogical strategies, biologists can also apply symmetrization procedures to organisms that are not genealogically close, as, for instance, the fact of considering the allometric relationships among mammals, choose experimental conditions that reduce the effects of their diversity.

^6^There are several theoretical scenarios in which such functionally significant variations can appear. One possibility consists of a significant geometrical change (as neovasculogenesis in the case of the vascular system) or a mutation (in the case of DNA) affecting a constraint. There are other scenarios, which include more general changes of organization (Montévil, 2019b), or the accumulation of small variations generating a massive and irreversible change. In all these situations, and under the hypothesis that they are not lethal, variations would induce a shift toward a different functional regime.

^7^The necessity of this justification makes a principled difference from biophysical relational models discussed above. While the latter can focus on some local constraints or constraints dependencies and could acknowledge that, “somehow,” these local phenomena are connected to the global organization, organizational models cannot focus on local phenomena if they cannot exhibit and justify the connection between the parts and the whole.

^8^Note that we write that “almost” all other cells depend on oxygen transport. χ refers here to the historical identity of organisms (they are mammals), and, as discussed, it can include variations. In cancers, for example, cells switch to the glycolytic metabolism that does not require oxygen, a phenomenon called “the Warburg effect” (Vander Heiden et al., 2009).

^9^Note that the genealogical specification of χ may also be more restrictive.

## References

[B1] Abolins S. R. Pocok M. Hafalla J. Riley E. Viney M. (2010). Measures of immune function of wild mice, *Mus musculus*. Mol. Ecol. 20, 881–892. 10.1111/j.1365-294X.2010.04910.x 21073587

[B2] Agutter P. Wheatley D. (2004). Metabolic scaling: consensus or controversy? Theor. Biol. Med. Model. 1:13 10.1186/1742-4682-1-13 15546492 PMC539293

[B3] Baker M. (2016). 1,500 scientists lift the lid on reproducibility. Nature 533, 452–454. 10.1038/533452a 27225100

[B4] Barandiaran X. Moreno A. (2008). Adaptivity: from metabolism to behavior. Adapt. Behav. 16, 325–344. 10.1177/1059712308093868

[B5] Beatty J. (1995). “The evolutionary contingency thesis,” in Conceptual Issues in Evolutionary Biology, eds G. Wolters and J. Lennox (Pittsburgh, PA: University of Pittsburgh Press), 45–81.

[B6] Boniolo G. Testa G. (2012). The identity of living beings, epigenetics, and the modesty of philosophy. Erkenntnis 76, 279–298. 10.1007/s10670-011-9308-9

[B7] Bookstein F. (2009). Measurement, explanation, and biology: lessons from a long century. Biol. Theory 4, 6–20. 10.1162/biot.2009.4.1.6

[B8] Canguilhem G. (1972). Le Normal et le Pathologique. Presses universitaires de France.

[B9] Chia R. Achilli F. Festing M. Fisher E. (2005). The origins and uses of mouse outbred stocks. Nat. Genet. 37:1181. 10.1038/ng1665 16254564

[B10] CZN International (1999). International Code of Zoological Nomenclature. London: International Trust for Zoological Nomenclature.

[B11] de Queiroz K. (1992). Phylogenetic definitions and taxonomic philosophy. Biol. Philos. 7, 295–313. 10.1007/BF00129972

[B12] Difrisco J. Mossio M (In press). “Diachronic identity in complex life cycles: an organizational perspective,” in Biological Identity: Perspectives from Metaphysics and the Philosophy of Biology, (History Philosophy of Biology), eds A. S. Meincke J. Dupré (New York, NY: Routledge).

[B13] Douady S. Couder Y. (1996). Phyllotaxis as a dynamical self organizing process part i: the spiral modes resulting from time-periodic iterations. J. Theor. Biol. 178, 255–273. 10.1006/jtbi.1996.0024

[B14] Festing M. (2014). Evidence should trump intuition by preferring inbred strains to outbred stocks in preclinical research. ILAR J. 55, 399–404. 10.1093/ilar/ilu036 25541542

[B15] Fleury V. (2009). Clarifying tetrapod embryogenesis, a physicist's point of view. Eur. Phys. J. Appl. Phys. 45, 1–54. 10.1051/epjap/2009033

[B16] Gilbert S. F. Sapp J. Tauber A. I. (2012). A symbiotic view of life: we have never been individuals. Q. Rev. Biol. 87, 325–341. 10.1086/668166 23397797

[B17] Godfray H. C. J. Knapp S. Mace G. M. (2004). The role of taxonomy in species conservation. Philos. Trans. R. Soc. Lond. Ser. B 359, 711–719. 10.1098/rstb.2003.1454 15253356 PMC1693347

[B18] Grandcolas P. (2017). Loosing the connection between the observation and the specimen: a by-product of the digital era or a trend inherited from general biology? Bionomina 12, 57–62. 10.11646/bionomina.12.1.7

[B19] Grimaldi D. A. Engel M. S. (2007). Why descriptive science still matters. BioScience 57, 646–647. 10.1641/B570802

[B20] Hernández I. Vecchi D. (2019). The interactive construction of biological individuality through biotic entrenchment. Front. Psychol. 10:2578. 10.3389/fpsyg.2019.02578 31849738 PMC6900962

[B21] Huggett N. Hoefer C. (2018). “Absolute and relational theories of space and motion,” in The Stanford Encyclopedia of Philosophy, ed E. N. Zalta (Metaphysics Research Lab; Stanford University). Available online at: https://plato.stanford.edu/archives/win2018/entries/spacetime-theories

[B22] Isaacs J. T. (1986). Genetic control of resistance to chemically induced mammary adenocarcinogenesis in the rat. Cancer Res. 46, 3958–3963. 3089584

[B23] Kauffman S. A. (1993). The Origins of Order: Self Organization and Selection in Evolution. New York, NY: Oxford University Press.

[B24] Kauffman S. A. (2019). A World Beyond Physics: The Emergence and Evolution of Life. New York, NY: Oxford University Press.

[B25] Lecointre G. (2015). Descent (Filiation). Dordrecht: Springer Netherlands.

[B26] Lecointre G. (2018). “The Boxes and their Content: What to Do with Invariants in Biology?” in Life Sciences, Information Sciences eds T. Gaudin, D. Lacroix, M. C. Maurel and J. C. Pomerol (London: John Wiley & Sons, Ltd), 139-152. 10.1002/9781119452713.ch14

[B27] Lecointre G. Le Guyader H. (2006). The Tree of Life: a Phylogenetic Classification, Vol. 20. Cambridge, MA: Harvard University Press.

[B28] Longo G. (2018). How future depends on past and rare events in systems of life. Found. Sci. 23, 443–474. 10.1007/s10699-017-9535-x

[B29] Longo G. Montévil M. Kauffman S. (2012). “No entailing laws, but enablement in the evolution of the biosphere,” in Genetic and Evolutionary Computation Conference, ed T. Soule (New York, NY: ACM), 1379–1392. 10.1145/2330784.2330946

[B30] Miquel P.-A. Hwang S.-Y. (2016). From physical to biological individuation. Prog. Biophys. Mol. Biol. 122, 51–57. 10.1016/j.pbiomolbio.2016.07.002 27431501

[B31] Mogil J. S. Wilson S. G. Bon K. Lee S. E. Chung K. Raber P.. (1999). Heritability of nociception i: responses of 11 inbred mouse strains on 12 measures of nociception. Pain 80, 67–82. 10.1016/S0304-3959(98)00197-3 10204719

[B32] Montévil M. (2019a). Measurement in biology is methodized by theory. Biol. Philos. 34:35 10.1007/s10539-019-9687-x

[B33] Montévil M. (2019b). Possibility spaces and the notion of novelty: from music to biology. Synthese 196, 4555–4581. 10.1007/s11229-017-1668-5

[B34] Montévil M. (in press). Historicity at the heart of biology. Theory Biosci. 10.1007/s12064-020-00320-8 32613275

[B35] Montévil M. Mossio M. (2015). Biological organisation as closure of constraints. J. Theor. Biol. 372, 179–191. 10.1016/j.jtbi.2015.02.029 25752259

[B36] Montévil M. Mossio M. Pocheville A. Longo G. (2016). Theoretical principles for biology: variation. Prog. Biophys. Mol. Biol. 122, 36–50. 10.1016/j.pbiomolbio.2016.08.005 27530930

[B37] Moreno A. Mossio M. (2015). Biological Autonomy. A Philosophical and Theoretical Enquiry. Dordrecht: Springer.

[B38] Mossio M. Bich L. (2014). “La circularité biologique: concepts et modéles,” in Modéliser & Simuler. Epistémologies et Pratiques de la Modélisation et de la Simulation, Vol. 2, eds Varenne, F., Silberstein, M., Huneman, P., and Dutreuil, S., editors (Paris: Éditions Matériologiques), 137–170.

[B39] Mossio M. Montévil M. Longo G. (2016). Theoretical principles for biology: organization. Prog. Biophys. Mol. Biol. 122, 24–35. 10.1016/j.pbiomolbio.2016.07.005 27521451

[B40] Mossio M. Pontarotti G. (2019). Conserving functions across Generations: heredity in light of biological organization. Br. J. Philos. Sci. axz031. 10.1093/bjps/axz031

[B41] Mossio M. Saborido C. Moreno A. (2009). An organizational account of biological functions. Br. J. Philos. Sci. 60, 813–841. 10.1093/bjps/axp036

[B42] Noonan H. Curtis B. (2018). “Identity,” in The Stanford Encyclopedia of Philosophy, ed E. N. Zalta (Metaphysics Research Lab; Stanford University). Available online at: https://plato.stanford.edu/entries/identity/

[B43] Nunes-Neto N. Moreno A. El Hani C. (2014). Function in ecology: an organizational approach. Biol. Philos. 29, 123–141. 10.1007/s10539-013-9398-7

[B44] Rashevsky N. (1954). Topology and life: in search of general mathematical principles in biology and sociology. Bull. Math. Biophys. 16, 317–348. 10.1007/BF02484495

[B45] Rosen R. (1991). Life Itself: A Comprehensive Inquiry Into the Nature, Origin, and Fabrication of Life. New York, NY: Columbia Univesrity Press.

[B46] Vander Heiden M. G. Cantley L. C. Thompson C. B. (2009). Understanding the warburg effect: the metabolic requirements of cell proliferation. Science 324, 1029–1033. 10.1126/science.1160809 19460998 PMC2849637

[B47] Varela F. Maturana H. Uribe R. (1974). Autopoiesis: the organization of living systems, its characterization and a model. Biosystems 5, 187–196. 10.1016/0303-2647(74)90031-8 4407425

[B48] Waters C. K. (2007). Causes that make a difference. J. Philos. 104, 551–579. 10.5840/jphil2007104111

[B49] Webster G. Goodwin B. (1996). Form and Transformation: Generative and Relational Principles in Biology. Cambridge, UK: Cambridge University Press.

[B50] West-Eberhard M. J. (2003). Developmental Plasticity and Evolution. Oxford, UK: Oxford University Press.

